# The use of tocilizumab in a child with systemic onset juvenile idiopathic arthritis and ganglioneuroblastoma

**DOI:** 10.1186/1546-0096-9-S1-P204

**Published:** 2011-09-14

**Authors:** Ristic Goran, Djokic Dragutin, Pasic Srdjan

**Affiliations:** 1 Department of Clinical Immunology and Pediatric Rheumatology, Department of Hematoncology, Mother and Child Health Institute of Serbia, Belgrade

## 

We report a case of a 6 year old girl with systemic onset juvenile idiopathic arthritis which presented at age of 3 years refractory to disease-modifying antirheumatic drugs and anakinra. During the first hospitalization a ganglioneuroblastoma (GNB) was diagnosed and consequently treated. During the SFOP treatment protocol for GNB our patient continued with low grade fever, but soon after the treatment the symptoms got worse, with significant rise of inflammation parameters, fever, rash and arthritis. The possibility of a GNB was immediately ruled out. During the next two years she received treatment with systemic corticosteroids, metotraxate, anakinra and thalidomide, but without any improvement or only partial with thalidomide. During this time, she also needed three times intraarticular corticosteroid treatment. We began treatment with a humanized monoclonal antibody against the interleukin-6 receptor, tocilizumab, which during the next six months resulted in almost complete remission of systemic symptoms (rash and fever), a fall in erythrocyte sedimentation rate, C-reactive protein, and ferritin serum levels, with graduate recovery in disability index and improvement of arthritis. Since her last course, she remains stable.

